# Transcription of *HOX* Genes Is Significantly Increased during Neuronal Differentiation of iPSCs Derived from Patients with Parkinson’s Disease

**DOI:** 10.3390/jdb11020023

**Published:** 2023-05-25

**Authors:** Viya B. Fedoseyeva, Ekaterina V. Novosadova, Valentina V. Nenasheva, Lyudmila V. Novosadova, Igor A. Grivennikov, Vyacheslav Z. Tarantul

**Affiliations:** Institute of Molecular Genetics of National Research Centre “Kurchatov Institute”, Moscow 123182, Russia; novek-img@mail.ru (E.V.N.); val-nenasheva1@yandex.ru (V.V.N.); novosadova-l@rambler.ru (L.V.N.); grivigan@mail.ru (I.A.G.); ninaslava130@yandex.ru (V.Z.T.)

**Keywords:** Parkinson’s disease, induced pluripotent stem cells, neural precursor cells, terminally differentiated neurons, RNA-seq, *HOX* genes

## Abstract

Parkinson’s disease (PD) is the most serious movement disorder, but the actual cause of this disease is still unknown. Induced pluripotent stem cell-derived neural cultures from PD patients carry the potential for experimental modeling of underlying molecular events. We analyzed the RNA-seq data of iPSC-derived neural precursor cells (NPCs) and terminally differentiated neurons (TDNs) from healthy donors (HD) and PD patients with mutations in *PARK2* published previously. The high level of transcription of *HOX* family protein-coding genes and lncRNA transcribed from the *HOX* clusters was revealed in the neural cultures from PD patients, while in HD NPCs and TDNs, the majority of these genes were not expressed or slightly transcribed. The results of this analysis were generally confirmed by qPCR. The *HOX* paralogs in the 3′ clusters were activated more strongly than the genes of the 5′ cluster. The abnormal activation of the *HOX* gene program upon neuronal differentiation in the cells of PD patients raises the possibility that the abnormal expression of these key regulators of neuronal development impacts PD pathology. Further research is needed to investigate this hypothesis.

## 1. Introduction

Parkinson’s disease (PD) is a common progressive neurodegenerative disease characterized by the death of dopaminergic (DA) neurons in the substantia nigra. This disease is associated with age and is one of the significant causes of disability of elderly people in modern society [1]. PD is a multifactorial neurodegenerative disorder in which a combination of environmental (lifestyle, dietary, infectious disease) and genetic factors make-up plays a role. Genetic factors are thought to play a major role in influencing endosomal, lysosomal, and mitochondrial dysfunction in PD pathophysiology. The molecular pathogenesis of PD involves a variety of mechanisms, including α-synuclein proteostasis, oxidative stress, mitochondrial dysfunction, apoptosis, autophagy, mitophagy, neuroinflammation, and epigenetic regulation [2,3]. Based on the experimental data available to date, many hypotheses about the mechanisms leading to PD have been proposed [4]. However, the pathogenesis of PD remains incompletely understood. 

The limited availability of material from PD patients is one of the problems when investigating the molecular mechanisms of this disease. One of the most promising approaches to solving this problem is the use of induced pluripotent stem cell (iPSC) technology [5,6,7,8,9,10,11]. The neural precursor cells (NPCs) and terminally differentiated neurons (TDNs) obtained by directed differentiation of iPSCs from PD patients provide an opportunity to study many aspects of PD phenotypes.

Among the molecular methods used to study PD mechanisms, high-throughput transcriptome analysis using next-generation RNA sequencing (RNA-Seq) is considered one of the most informative. This method makes it possible to obtain data on differential gene expression in norm and pathology, which facilitates the identification of genes directly or indirectly involved in the pathological process.

We have previously performed the full-transcriptome analysis of gene expression in iPSC-derived NPCs and TDNs from three PD patients and three healthy donors (HD) using RNA-seq [12]. Among the top 10 upregulated differentially expressed genes (DEGs) in NPCs and TDNs from two PD patients compared to HD, we observed the increased expression of many *HOX* genes that were almost not expressed in cells from HD. We could not find any data in the existing literature on the presence of any relationship between *HOX* genes and PD.

Mammalians have 39 *HOX* genes organized in four clusters: *HOXA* (7p15), *HOXB* (17q21.2), *HOXC* (12q13), and *HOXD* (2q31). *HOX* loci are characterized by the presence of both protein-coding and non-coding genes [13] with synchronously regulated expression. HOX proteins are the transcription factors. They can bind to promoter or enhancer regions of target genes, alone or as members of the HOXasomes, in order to activate or repress target gene transcription. Some microRNAs and long non-coding RNAs (lncRNAs), which are encoded in *HOX* clusters, target both *HOX* and other genes [13,14,15,16,17,18].

It is well known that under normal conditions, the main activity of *HOX* gene clusters occurs during the embryonic period of development. The 3′–5′ order of the *HOX* genes within the clusters typically correlates with their temporal and spatial expression along the embryonic anterior-posterior axis, which is associated with the existence of common regulatory elements [19]. Region-specific *HOX* gene expression was shown along the rostrocaudal and dorsoventral axes at the early stages of neuronal development. However, their expression during late embryonic and postnatal stages of the nervous system has been poorly characterized.

The expression of *HOX* genes is terminated in most cells of the adult organism. In particular, there are indications that *HOX* gene expression is essentially absent in healthy human adult brains [20]. It should be noted that there is evidence regarding *HOX* gene expression in individual cells in adulthood, suggesting that they may continue to play a role in cellular identity for tissue maintenance and stem cell renewal [21]. There is also evidence that the *HOX* transcription factors can regulate synaptogenesis processes even after the process of axonal and dendritic guidance [22]. The mutations in 10 out of 39 *HOX* genes in humans may be associated with 15 genetic disorders [23]. Abnormal high expression of individual *HOX* genes was found in various tumors, including those associated with breast cancer [24] and brain tumors [20]. Some *HOX* cluster-embedded non-coding RNAs demonstrated crucial regulatory functions in development, adult homeostasis, and cancer [25,26].

In this study, the results of gene transcription analysis of *HOX* clusters in iPSC-derived NPCs and TDNs from PD patients compared with corresponding HD cells were presented. Our data indicated that the expression of many protein-coding and non-coding genes of *HOX* clusters in the cells of PD patients was turned on during neuronal differentiation of iPSC, while they were almost completely turned off in the cells of HD.

## 2. Materials and Methods

### 2.1. Generation of Human iPSC-Derived NPCs 

All cell lines were subjected to procedures in accordance with the standard differentiation protocol (Figure S1). iPSCs were cultured in CO_2_ incubator (5% CO_2_, 80% humidity, and 37 °C) in iPS-Brew XF basal medium (Miltenyi Biotec, Nordrhein-Westfalen, Germany) until reaching an 80% confluent monolayer. The culture medium was replaced by the medium for neural precursors: Neurobasal medium (Gibco, Carlsbad, CA, USA), penicillin–streptomycin (50 U/mL; 50 µg/mL) (Paneco, Moscow, Russia), 2% serum replacement (Gibco, Carlsbad, CA, USA), 1% N2 (Life Technologies, Carlsbad, CA, USA), 2 mM L-glutamine (ICN Biomedicals Inc., Hackensack, NJ, USA), 1 mM non-essential amino acids (Paneco, Moscow, Russia), 10 μM SB431542 (Stemgent, Cambridge, MA, USA), and 80 ng/mL recombinant Noggin (Peprotech, Cranbury, NJ, USA). During the next 14 days, the medium was changed every other day. On the 15th day of cultivation, the cells were collected for the analyses (qPCR and RNA-seq) or stained with the neural cell markers. After reaching the monolayer, NPCs were detached with 0.05% trypsin and then plated on new Petri dishes coated with Matrigel (Corning Life Sciences, Corning, NY, USA) at a dilution of 1:4 or 1:5. Cells were cultured in a CO_2_ incubator (5% CO_2_, 80% humidity, and 37 °C). The characteristic immunocytochemical staining of NPCs (Figure S2) revealed no significant differences between cell lines from PD patients and HD (a detailed description of the method is given in Section 2.3).

### 2.2. Targeted Differentiation of NPCs into TDNs

The NPCs were disseminated at 200,000 cells per cm^2^ into Petri dishes pretreated with Matrigel in neuronal precursor medium supplemented with 5 µM Rock (StemoleculeY27632, Stemgent, Cambridge, MA, USA). The next day, the medium was replaced with medium for the differentiation of type I neurons (Neurobasal medium A, (Gibco, Carlsbad, CA, USA), penicillin–streptomycin (50 U/mL; 50 µg/mL) (Paneco, Moscow, Russian Federation), 2% serum replacement (Gibco, Carlsbad, CA, USA), 1% B-27 (Life Technologies, Carlsbad, CA, USA), 2 mM L-glutamine (ICN Biomedicals Inc., Hackensack, NJ USA), 1 mM non-essential amino acids (Paneco, Moscow, Russia), 100 ng/mL human SHH (Miltenyi Biotec, Nordrhein-Westfalen, Germany), 100 ng/mL FGF8 (PeproTech, Cranbury, NJ, USA), and 10 μM purmorphamine (Sigma-Aldrich, Saint Louis, MO, USA). The cells were cultured for 10 days (15–26 days from the beginning of differentiation), with the medium changing every other day. After the cells reached a dense monolayer, they were disseminated to new 1:4 or 1:5 cups. On the ninth day of cultivation, the cells were detached with 0.05% trypsin (Gibco, Carlsbad, CA, USA) and disseminated on a prepared culture dish at 400,000 cells per cm^2^ in the medium for the differentiation of type I neurons with the addition of 5 µM Rock (StemoleculeY27632, Stemgent, Cambridge, MA, USA). The next day, the medium was changed to medium for the differentiation of type II neurons (Neurobasal medium A, (Gibco, Carlsbad, CA, USA), penicillin–streptomycin (50 U/mL; 50 µg/mL) (Paneco, Moscow, Russia), 2% serum replacement (Gibco, Carlsbad, CA, USA), 1% B-27 (Life Technologies, Carlsbad, CA, USA), 2 mM L-glutamine (ICN Biomedicals Inc, Hackensack, NJ, USA), 1 mM non-essential amino acids (Paneco, Moscow, Russia), 20 ng/mL BDNF (PeproTech, Cranbury, NJ, USA), 20 ng/mL GDNF (PeproTech, Cranbury, NJ, USA), 200 μM ascorbic acid (StemCell, Vancouver, BC, USA), and 4 μM Forskolin (Stemgent, Cambridge, MA, USA)) and the cells were cultured for 14 days (27–45 days from the beginning of differentiation). The medium was changed every other day for the first 7 days and daily thereafter. Cells were cultured in CO_2_-incubator (5% CO_2_, 80% humidity, and 37 °C). At the end of differentiation at the stage of TDNs (45 days of differentiation), the cells were stained with the neural cell markers and were collected for RNA isolation and subsequent analyses (qPCR and RNA-seq) (the methods are described below). The characteristic immunocytochemical staining of TDNs with antibodies against β III tubulin and tyrosine hydroxylase (TH) was shown in Figure S3. This staining revealed no significant differences between cell lines from PD patients and HD.

Additionally, we compared the expression levels of the neuronal marker genes in NPCs and TDNs from PD patients and HD, which were obtained by RNA-seq analysis as normalized read counts (NRC) per geometric mean of the four most stable housekeeping genes GAPDH, RPLP0, PPIA, β-ACTIN identified by the method [27] adapted for RNA-seq data analysis (Table S1). The ratios of transcription levels of genes-markers of the different stages of neuronal differentiation in TDNs and NPCs (TDNs NRC/NPCs NRC) show the degree of differentiation in the cells of healthy donors and patients with PD. The data demonstrated similar levels of the many marker genes both in cells of patients with PD and HD. The increased expression of neuronal markers (*TUBB3*, *NEUN*, *MAP2*, *MSI1*) and the markers of synaptogenesis (*SNAP25*, *SYT1*, *PSD95*, *RAB5A*) was observed in TDN from both PD patients and HD compared with NPC, which indicated the acquisition of a mature phenotype by cells. During the neuronal differentiation, the increase of the expression of marker gene *NURR1* of DA neurons was observed in TDNs from HD and PD patients (additionally to TH immunostaining (Figure S3). Moreover, the gene set enrichment analysis (GSEA, described in 2.4) of DEGs obtained when comparing HD TDNs and NPCs (Table S2) or PD TDNs and NPCs (Table S3) showed similar changes in many categories, associated with the appearance of properties of mature neurons. 

Summarizing, based on immunostaining and RNA-seq data, we can conclude that neuronal differentiation of iPSCs from both PD patients and HD occurred in a similar manner up to the TDN stage.

### 2.3. Immunofluorescence Staining

On the 15th and 45th day of neuronal differentiation, cells were washed with PBS, fixed with 4% para-formaldehyde in PBS (pH 6.8) for 20 min at room temperature (RT), and then washed in PBS with 0.1% Tween 20 (Sigma-Aldrich, Saint Louis, MO, USA) three times for 5 min. Nonspecific antibody sorption was blocked by incubation in blocking buffer (PBS with 0.1%, Triton X-100, and 5% fetal bovine serum (HyClone, Waltman, MA, USA)) for 30 min at RT. Primary antibodies Rabbit anti-tyrosine hydroxylase (TH), Mouse anti-βIII Tubulin, and Rabbit anti—Sox1 (all from Abcam, Cambridge, UK) were applied overnight at 4 °C and then washed in PBS with 0.1% Tween 20 three times for 5 min. The secondary antibodies Goat anti-Rabbit IgG (H + L), AF546, Goat anti- Mouse IgG (H + L), AF488 (all from ThermoFisher, Waltham, MA, USA), were applied for 60 min at RT, then washed in PBS with 0.1% Tween 20 three times for 5 min. After that, the cell cultures were incubated with 0.1 μg/mL DAPI (Sigma-Aldrich, Saint Louis, MO, USA) in PBS for 10 min for visualization of the cell nuclei and washed twice with PBS. The cells were investigated using an AxioImager Z1 fluorescence microscope (Carl Zeiss, Oberhohen, Germany), and images were taken with AxioVision 4.8 software (Carl Zeiss, Oberhohen, Germany).

### 2.4. Bioinformatic Analysis

In the previous study [12], we published transcriptome profiles of NPCs and TDNs differentiated from iPSCs of 3 HD and 3 patients with PD carrying different mutations in the *PARK2* gene which were obtained according to the previously described technique [4,8,28]. The datasets from [12] containing raw sequence data from triplicate biological replicates were converted to FASTQ format. Raw transcriptome sequence reads were deposited in Gene Expression Omnibus (GEO) series GSE181029. Paired reads trimming for quality and quantification against Homo Sapiens GRCh38.13 genome annotation was carried out as outlined in the previous work [12].

**Figure 1:**
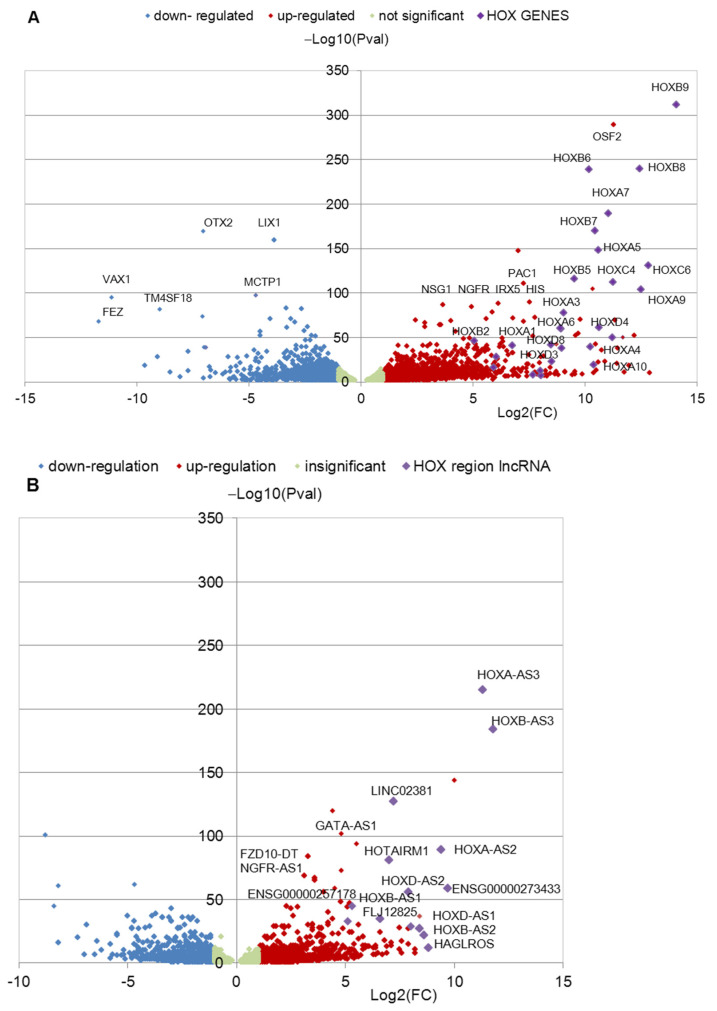
Volcano plot demonstrating an overview of the differential expression of protein-coding (**A**) and non-coding (**B**) genes in NPCs from the PD2 and PD3 groups compared with mean value of the expression of genes in HD NPCs. Region of insignificant DEG corresponds to |Log_2_(FC)| < 1 and FDR < 0.05. In total, (**A**) 4883 genes are presented; (**B**) 3530 long non-coding RNAs are presented.

**Figure 2:**
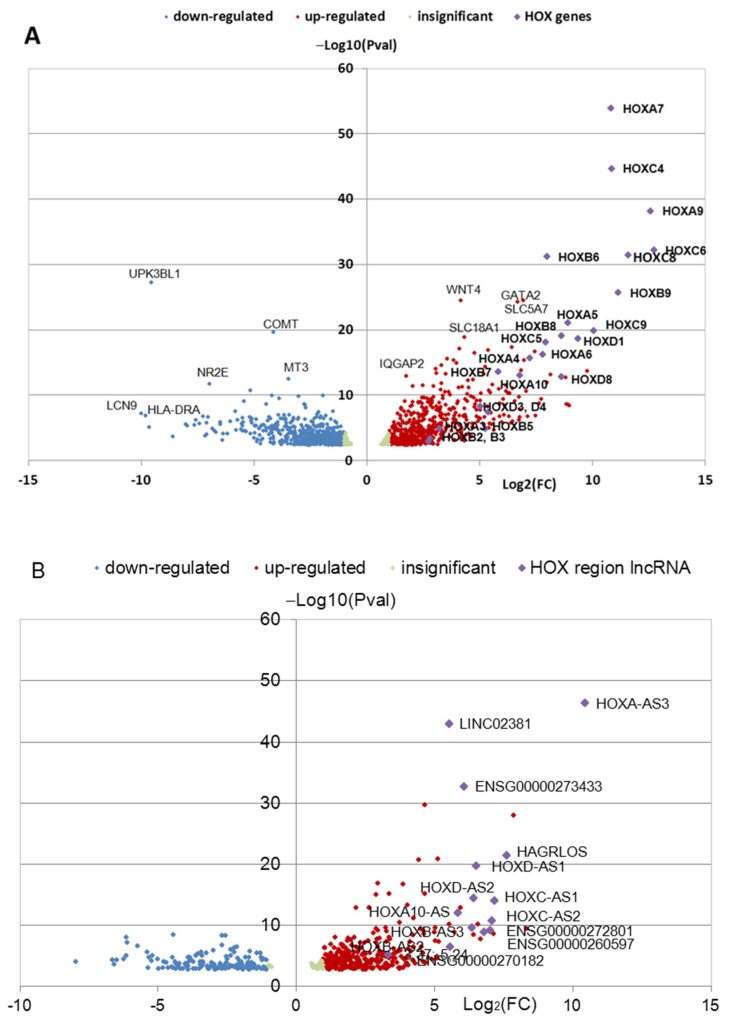
Volcano plot demonstrating an overview of the differential expression of protein-coding (**A**) and non-coding (**B**) genes in TDNs from the PD2 and PD3 groups compared with mean value of the expression of genes in HD TDNs. Region of insignificant DEG corresponds to |Log_2_(FC)| < 1 and FDR < 0.05. In total, (**A**) 1224 genes are presented; (**B**) 494 long non-coding RNAs are presented.

Data on differential gene expression were obtained using the “edgeR” package [29]. Read counts were normalized using TMM algorithm in the R-package “edgeR” [29], and the values of counts per million (CPM) > 1.5 for coding gene transcripts and CPM > 0.1 for lncRNA were chosen. DEGs were selected based on the fold change threshold |log2(FC)| > 1.0, false discovery rate (FDR) < 0.05, and Pval < 0.05. Gene set enrichment analysis (GSEA) of DEGs data was performed using programs available on http://WebGestalt.org server (accessed on 14 January 2019) [30]. The categories “Biological Process” and “Molecular Function” from Gene Ontology (GO), Hallmark50, pathway data base KEGG, and others were useful for the analysis of DEG clusters from the multitude of data bases of this server. The gene names and identification numbers (ID in Ensembl Release 107 12 July 2022) of *HOX* cluster genes used in our investigation were presented in Table S4. Volcano Plots for the genes were created by MS Office (Excel) to present DEGs (Figure 1 and Figure 2).

### 2.5. Real-Time PCR Analysis (qPCR)

Total RNA was isolated from cells using a Trizol RNA purification kit (Invitrogen, Waltham, MA, USA) according to the manufacturer’s recommendations, followed by DNA processing. cDNA was synthesized from 2 µg of total RNA using reverse transcriptase M-MLV (Evrogen, Moscow, Russia) with random primers. The obtained cDNA was amplified using the Roche LightCycler system. The reaction conditions were as follows: denaturation at 95 °C (3 min), followed by 40 cycles (95 °C, 15 s; 60–65 °C, 20 s; and 72 °C, 45 s). Reaction mixture qPCRmix-HS SYBR (Evrogen, Moscow, Russia) was used. To confirm amplification specificity, qPCR products were subjected to melting curve analysis, in which only one peak was observed. Crossing point values were measured using LightCycler version 2.0 analysis software (Roche, Basel, Switzerland) and the final quantification was performed using the comparative 2^∆Ct^ method [31]. Three repeated reactions were performed for each gene analyzed, and the values were normalized to 18S rRNA. All primers for qPCR were designed using NCBI Primer-BLAST (Table S5). Results were presented as the mean ± SD of at least three independent experiments. Quantification was performed by measuring the value of the quantification cycle (Ct). Target gene levels in each sample were normalized to *18S rRNA* using the following formula:

Gene expression level = 2^Ct(18S rRNA)-Ct(HOX)^ = 2^∆Ct^

### 2.6. Statistical Analysis

For the detection of read counts normality, we used Shapiro–Wilk test. In the case of read counts with normal distribution, the Welch *t*-test was used to compare data obtained from cells of PD patients and HD. In the cases of genes without data normality, Mann–Whitney U-test was used to find out the validation of differences. Normalized read counts of neuronal marker genes were analyzed by the ANOVA test and Mann–Whitney U-test.

## 3. Results

### 3.1. Identification of Differentially Expressed HOX Cluster Genes in NPCs and TDNs from PD Patients

**Figure 3:**
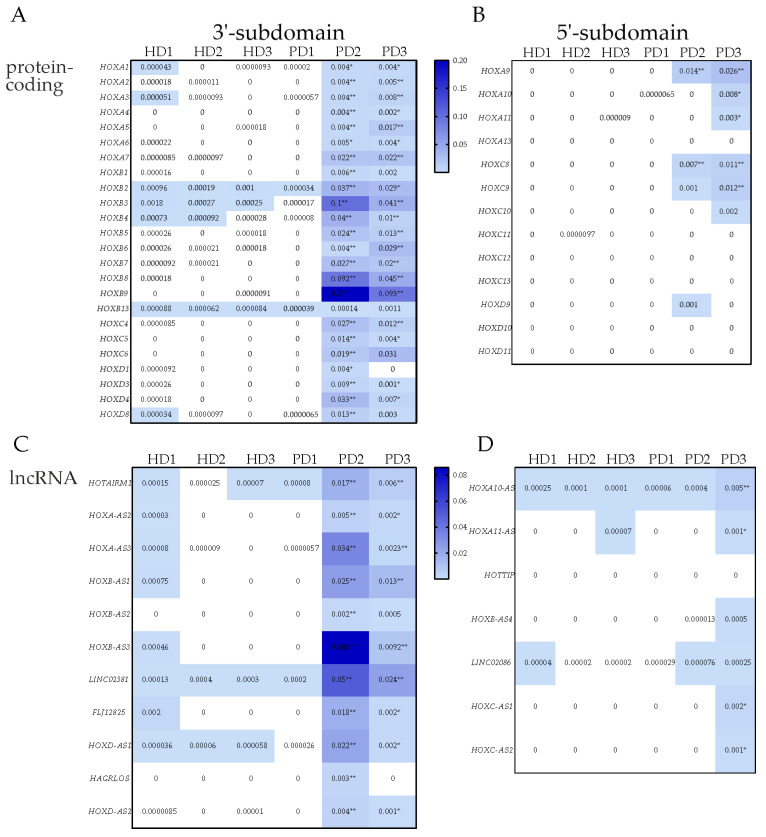
Heatmap of the transcription of protein-coding and lncRNA genes of *HOX* clusters in iPSC-derived NPCs. Expression of (**A**,**B**) protein-coding HOX genes or (**C**,**D**) lncRNA genes in NPCs calculated as NRC. The order of the genes corresponds to their location in the 3′ to 5′ direction in the cluster. Statistical analysis was performed for groups PD1, PD2, and PD3 in comparison with HD group (mean of HD1, HD2, and HD3) (all in three biological replicates). Mann–Whitney *U*-test * *p* < 0.05, Welch *t*-test ** *p* < 0.01.

**Figure 4:**
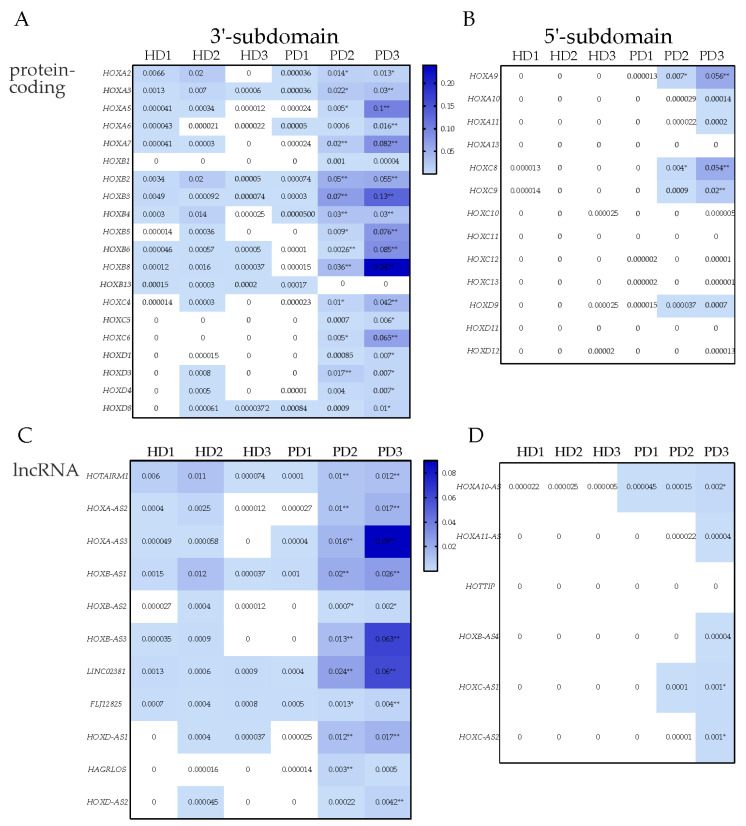
Heatmap of transcription of protein-coding and lncRNA genes of *HOX* clusters in iPSC-derived TDNs. Expression of protein-coding *HOX* genes (**A**,**B**) or lncRNA genes (**C**,**D**) in TDNs of PD patients and HD was calculated as NRC. The order of the genes corresponds to their location in the 3′ to 5′ direction in the cluster. Statistical analysis was performed for groups PD1, PD2, and PD3 in comparison with HD group (mean of HD1, HD2, and HD3) (all in three biological replicates). Mann–Whitney U-test * *p* < 0.05, Welch *t*-test ** *p* < 0.01.

The iPSC cell lines used in the study are presented in Table 1. The iPSC cell lines from the three healthy donors and three patients with PD were differentiated into NPC and TDN, and RNA-seq analysis of the obtained cultures in triplicate was performed as described in [12]. Bioinformatic analysis of raw transcriptome sequence reads, which were deposited in GEO series GSE181029, revealed DEGs in PD NPCs and TDNs compared to HD cells. The volcano plots (Figure 1 and Figure 2) demonstrate the distribution of DEGs among the top 100 genes in NPCs (Figure 1A,B) and in TDNs (Figure 2A,B). There are a number of *HOX* cluster’ genes (coding and non-coding proteins) among the top 100 genes upregulated in patients PD2 and PD3 DEGs with the lowest FDR and Pval values in NPCs and TDNs from two PD patients compared to HD (Figure 1 and Figure 2). It should be noted that in the cells from PD1 patients, the upregulation of *HOX* gene expression was not found in the same way as in the cells from HD (Figure 3 and Figure 4).

Additionally, GSEA for NPCs and TDNs from PD2 and PD3 patients versus HD cells showed the enrichment of the next categories in GO resource: “Biological Process” (GO: 0048658 “Embryonic development”, GO: 0021675 “Nerve development”, GO: 0048705 “Skeleton system morphogenesis”), and “Molecular Function” (GO: 0035326 “Enhancer binding”, GO:0033613 “Activating Transcription factor binding”) in which leading positions were occupied by the *HOX* genes (Table S6). Each category had a high positive Normalized Enrichment Score (NES ~ 2.5) and a low FDR < 0.05 and Pval < 0.005. Therefore, we turned our attention to the *HOX* cluster genes and selected them for detailed consideration.

Our results for *HOX* cluster were further presented in two forms: (1) as normalized read counts (NRC) and (2) as transcripts per million (TPM).

### 3.2. Transcription of HOX Gene Clusters in NPCs Derived from PD Patients and HD

The data obtained by the bioinformatic analysis of RNA-seq on the transcription of protein-coding genes of *HOX* clusters in iPSC-derived NPC from PD patients and HD are shown in Figure 3A and Figure S4A. When assessed by NRC values, the significantly upregulated expression of *HOXA1*, *A2*, *A3*, *A4*, *A5*, *A6*, A7, and *A9* among *HOXA* cluster genes, *HOXB1*, *B2*, *B3*, *B4*, *B5*, *B6*, *B7*, *B8*, and especially *B9* in HOXB cluster, *HOXC4*, *C5*, *C6*, *C8*, *C9* genes in *HOXC* cluster, and *HOXD1*, *D3*, *D4*, *D8* genes was observed in iPSC-derived NPCs PD2 and PD3 (Figure 3A). In NPCs from both PD patients, the most significant quantitative increase in transcription was found for the *HOXA* and *HOXB* cluster genes (*HOXA3*, *A7*, *HOXB3*, *B8*, and especially *B9*). It should be noted that, in general, the results obtained with the NRC values assessment were similar to those obtained with the TPM assessment (Figure 3A and Figure S4A).

Thus, at the stage of NPC, the transcription of many genes of *HOX* clusters was enhanced in the cells PD2 and PD3 of PD patients, whereas these genes were only slightly expressed in cell lines HD and PD1 (Figure 3A). It should be noted that, in general, the results obtained indicate a more enhanced expression of 3′ *HOX* paralogs compared to 5′ *HOX* paralogs in the NPCs from PD patients (Figure 3B).

Along with protein-coding genes, there are genes encoding lncRNAs in *HOX* gene clusters that take part in the regulation of the formers. These genes were also slightly expressed in NPCs derived from HD (Figure 3C and Figure S4B). At the same time, their expression was turned on in the NPC lines from PD2 and PD3 patients. The increased level of transcription, as assessed by NRC, was observed for the *HOTAIRM1*, *HOXA-AS2*, *HOXA-AS3*, *HOXA10-AS* genes in the *HOXA* cluster, *HOXB-AS1*, *HOXB-AS2, HOXB-AS3* genes in the *HOXB* cluster, *LINC02381*, *FLJ12825*, *HOXC-AS1*, *HOXC-AS2* genes in the *HOXC* cluster, and for *HOXD-AS1*, *HAGLROS*, and *HOXD-AS2* in the *HOXD* cluster (Figure 3C). Additionally, in NPCs from PD patients, elevated levels of TPM of *AC004080.6* (cluster *HOXA*) and *RP11-834C11* (cluster *HOXC*) genes were found (Figure S4B). Thus, at the stage of NPC, the transcription of many genes encoding lncRNAs of *HOX* clusters was increased in the PD2 and PD3 NPCs compared with HD NPCs, similarly to HOX protein-encoding genes (the expression of the 3′-paralogs was increased to a greater extent compared to the 5′-paralogs (Figure 3C,D)).

### 3.3. Transcription of HOX Gene Clusters in TDNs Derived from PD Patients and HD

The *HOX* cluster protein-coding gene transcription data obtained by bioinformatic analysis of RNA-seq of iPSC-derived TDNs from PD2 and PD3 patients were demonstrated in Figure 4A and Figure S5A. In TDNs from HD and PD1 patients, transcription of most genes in *HOX* cluster was at a very low level (Figure 4). Only some of them, especially those belonging to the *HOXB* cluster, were transcribed in HD TDNs at the level exceeding the background. However, the expression of such genes was at a level much lower than in PD TDNs (Figure 4).

When evaluated by NRC, the significantly increased expression of *HOXA2*, *A4*, *A5*, *A7*, *A9* (*HOXA* cluster), *HOXB2*, *B3*, *B4*, *B5*, *B6*, *B8*, *B9* (*HOXB* cluster), *HOXC4*, *C6*, *C8*, *C9* (*HOXC* cluster), *HOXD3*, *D4*, *D8* (*HOXD* cluster) was observed in TDNs from PD2 and PD3 patients (Figure 4A). In addition, the *HOXA3* gene was upregulated in TDNs of PD patients when measured in TPM (Figure S5A). The transcription of all these genes was also increased in PD NPCs (Figure 3A). Similar to in PD NPCs, the highest level of expression was observed for the HOXB and HOXA cluster genes (especially *HOXB9*) in PD TDNs, supposing their important role in these cells.

In PD TDNs, as well as in PD NPCs, the increased transcription of many genes encoding lncRNAs was detected, while they were almost unexpressed in HD: *HOXA-AS2, HOTAIRM1*, *AC004080.6*, *HOXA-AS3*, *HOXA10-AS* lncRNA genes of *HOXA* cluster, *HOXB-AS1*, *HOXB-AS3* genes of *HOXB* cluster, *LINC02381* and *FJL12825* genes of *HOXC* cluster, and *HOXD-AS1* and *HOXD-AS2* genes of *HOXD* cluster (Figure 4C and Figure S5B). All these genes were also upregulated in PD2 and PD3 lines of NPCs (Figure 3C). The results obtained with the NRC were generally similar to those obtained with the TPM due to the small variation in the length of predominantly transcribed mRNA (Figure S5).

Summarizing the data on the transcription of protein-coding *HOX* genes and genes encoding lncRNAs in NPCs and TDNs, we could note that, in general, cell lines PD2 and PD3 obtained from the PD patients with the mutations in *PARK2* had similar activated expression profiles, while in HD NPCs and TDNs *HOX,* cluster genes were almost completely off or were transcribed at a very low level.

### 3.4. Real-Time PCR Analysis of HOX Cluster Gene Expression

At first, we evaluated the expression of randomly selected protein-encoding *HOX* genes in all four clusters in the fibroblasts used to obtain iPSCs from HD and PD patients and in the iPSCs themselves. The qPCR analysis demonstrated that *HOX* gene transcription in fibroblasts from PD patients and HD was at a similar level (Figure S6). At the same time, in iPSCs, obtained from these fibroblasts, transcription of *HOX* genes was undetectable at all (Figure S6), which was consonant with the available literature data [33].

**Figure 5:**
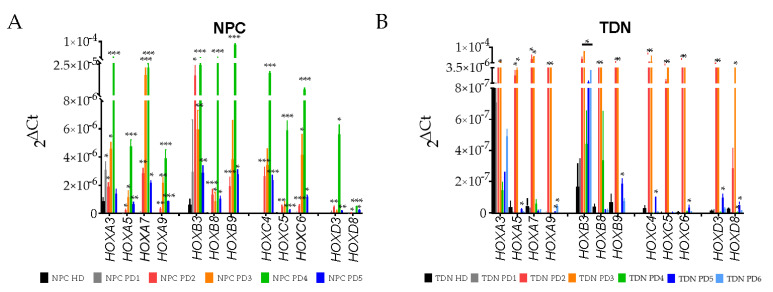
RT-qPCR analysis of *HOX* gene transcription. (**A**) NPCs and (**B**) TDNs from HD (average of HD1, HD2, HD3) (black), PD1 (grey), PD2 (red), PD3 (orange), PD4 (green), PD5 (dark blue), and PD6 (light blue) patients (all in three biological replicates). 18S rRNA was used as a reference gene. PD vs. HD. Benjamini-Hochberg correction for multiple comparisons was used. * *p* adj < 0.05, ** *p* adj < 0.01, *** *p* adj < 0.001.

To verify the data on the transcription obtained by transcriptome sequencing, we performed qPCR analysis for the selected *HOX* genes in NPC and TDN and demonstrated that the results of bioinformatic analysis of RNA-seq data were generally confirmed by qPCR (Figure 5, PD2 and PD3 samples bars are coloured by red and orange).

Next, we examined by qPCR the transcription of the same *HOX* genes in iPSC-derived NPCs and TDNs from PD patients with the mutations in other PD-associated gene lines PD4, PD5, and PD6 (Figure 5, green, dark blue, and light blue bars) and also observed the increased expression of protein-coding *HOX* genes in these cells compared to HD (Figure 5, black bars). It should be noted that in NPC of the majority of patients, the increase of HOX gene expression was observed, while in TDNs, the HOX activation was the characteristic presumably of PD2 and PD3 cells from patients with mutations in the *PARK2* gene. Nevertheless, when iPSC derived from PD patients with the mutations in different genes differentiating the HOX gene activation was observed and supposedly might be a characteristic hallmark of PD neural derivatives.

### 3.5. Differential Expression of the Activators of the HOX Genes in NPC and TDN from PD Patients

**Figure 6:**
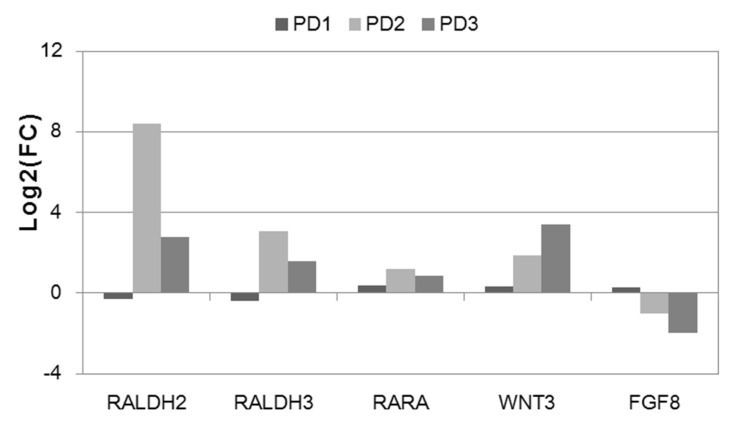
Differential expression of 3′*HOX* cluster activation genes *RALDH2*, *RALDH3*, *WNT3*, retinoic acid nuclear receptor *RARA*, and activation gene *FGF8* of 5′ *HOX* cluster in NPC PD1, PD2, and PD3 lines in comparison with NPC from HD (mean of HD1, HD2, HD3, RNA-seq data). FDR < 0.05, Pval < 0.05 for all gene expression in PD2 and PD3 cells vs. HDmean.

In order to find out the possible mechanism of the *HOX* gene activation, we analyzed the expression of the potential activators of *HOX* genes and found the upregulation of *RALDH2* and *RALDH3* genes (Figure 6) in NPC and TDN from PD2 and PD3 patients that encoding the main enzymes in the synthesis of retinoic acid (RA), which is an activator of expression of predominantly the 3′ region of *HOX* cluster genes [34,35,36,37,38]. We also found the enhanced expression of the RARα, the RA receptor, that can influence the Wnt/β-catenin pathway known to be involved in PD pathogenesis [39] (Figure 6). The activation of the WNT3 gene was also found in cells of PD2 and PD3 patients compared to HD NPC (Figure 6). At the same time, the gene encoding the transcriptional factor FGF8, which is an activator of the 5′ region of *HOX* cluster genes, was downregulated in PD cells vs. HD (Figure 6). This fact correlates with the less activation of the 5′-HOX genes during the differentiation of PD iPSC into NPCs and TDNs (Figure 3 and Figure 4).

## 4. Discussion

Bioinformatic analysis of transcriptome datasets obtained from NPCs and TDNs differentiated from iPSCs of HDs and PD patients with mutations in the *PARK2* gene [12] revealed a significant difference in the transcription of genes located in four *HOX* clusters. The expression of *HOX* cluster genes in the cells of two PD patients was significantly increased during neuronal differentiation of iPSC (in NPCs and TDNs, especially in NPCs), whereas in the cells of HD (both NPCs and TDNs), we found that the protein-coding genes and lncRNA-encoding genes located in *HOX* clusters were almost completely off or transcribed at a very low level (at least two orders of magnitude lower than in PD patients). Some *HOX* genes, especially those belonging to the *HOXB* cluster, were transcribed in NPCs and TDNs of HDs, but the expression levels were much lower than in PD2 and PD3 NPCs and TDNs (Figure 3 and Figure 4).

The data obtained from RNA-seq analysis were verified by qPCR (Figure 5). Moreover, the qPCR analysis of RNA in NPCs and TDNs from PD patients with other mutations (*GBA, PARK8,* and *GBA* + *PARK8*) (Figure 5) also showed the activation of *HOX* cluster genes, especially in NPCs, thus demonstrating the more general nature of this process. Additional analysis of our previously published data on DEGs in sporadic cases of PD in twins [40] showed the increased expression level of some *HOX* genes in NPCs from one more PD patient (Figure S7). However, it should be noted that such an association was not always observed. NPCs and TDNs derived from PD1 patient iPSC did not reveal *HOX* gene expression as well as a differential expression of *HOX* genes was not detected in the NPCs from one other patient with a sporadic case of PD in twins compared with that in HD (Figure S7 [40]). Thus, in neuronal cell lines obtained from 6 out of 8 PD patients, a significant increase in the expression of a number of *HOX* genes at the level of transcription was revealed.

The *HOX* clusters contain numerous non-coding protein genes that are involved in the regulation of the expression of protein-coding *HOX* genes. There is evidence that the expression of miRNAs from *HOX* cluster may be altered in neurodegenerative diseases [41]. It is also known that lncRNAs also can play an important role in neurological disorders [42], in particular in the pathogenesis of PD [43,44,45]. Our bioinformatic analysis revealed the massive activation of lncRNA genes located in *HOX* clusters in addition to protein-coding genes in NPCs and TDNs from PD patients compared to HD. Among these non-protein-coding genes, the *HOTAIRM1*, *HOXA-S3*, *HOXB-AS1*, *HOXB-AS3*, *LINC02381*, and *HOXD-AS1* genes had the highest levels of transcription.

It is known that the expression of *HOX* genes and lncRNA genes in *HOX* clusters during development is tightly regulated in a spatiotemporal manner, in part due to epigenetic processes. Two subdomains (3′ and 5′) were previously allocated in HOX gene clusters, including corresponding 3′- and 5′-parts of the *HOXA*, *C*, and *D* clusters [34,46] (Figure 3 and Figure 4). Within each part of the clusters, good coordination of the transcription of the genes encoding the proteins and lncRNA could be seen in PD2 and PD3. This was particularly evident for the *HOXA* and *HOXC* clusters. The co-activation of protein-coding genes and non-coding intergenic lncRNAs transcription in the *HOXA* cluster was also described earlier [43]. The authors concluded that these lncRNAs affected the distribution of repressor and activator tags in chromatin. In particular, there were data indicating the remodulating activity of *HOTAIRM1* in relation to repressive complexes [19]. HOTAIRM1 was shown to influence three-dimensional chromatin organization and, in this way, activate *HOXA* genes [47]. Consistent with these results, we found that high levels of *HOTAIRM1* transcription correlated with elevated levels of the *HOXA3* and *HOXA5* genes in the *HOXA* cluster in the NPCs from both patients (Figure 3C and Figure S4B).

It should be noted that the transcription of lncRNAs in *HOX* loci positively correlated with that of genes encoding *HOX* proteins belonging to the same subdomain and negatively correlated with the transcription of *HOX* genes located in another subdomain (Figure 3, Figure 4, Figure S4, and Figure S5) which suggested the presence of common features in regulation within each subdomain. In turn, we can see that the anti-sense RNA transcription that has complementary fragments to specific protein-coding genes can correlate both negatively and positively with the transcription of these genes. The possible suppressive function of anti-sense RNAs might be related to RNA interference in the case of co-localization with one or more transcriptional variants of a particular gene, while the activating function was probably related to its participation in the loop formation of chromatin. This assumption is based on the fact that many anti-sense RNAs have responsive elements for the insulator-binding protein CTCF as an essential component of the chromatin loop formation mechanism involving RNA [48,49]. Such responsive elements were largely presented in *HOXC-AS2* (Figure S8) [50]. The *HOXB-AS3*(0.523) and *HOXB-AS3*(0.522) transcript variants (Figure S4B) were co-localized with the *HOXB5* and *HOXB6* genes, and perhaps the less prominent increased expression of the *HOXB5* and *HOXB6* genes was related to this fact, whereas the *HOXB8* and *HOXB9* gene regions showed no anti-sense RNA according to annotated data and, namely, *HOXB8* and *HOXB9* genes had the highest level of transcription in the *HOXB* locus at the NPC and TDN stages in relation to PD patients.

RA is known as an activator of expression of predominantly the 3′ region of *HOX* cluster genes [33,34,35,36,37,38]. We found in our study that the *RALDH2* and *RALDH3* genes, which encode main enzymes in the synthesis of RA, had significantly higher levels of transcription (from 4 to 250 times) in the NPC in both patients with PD compared to HD (Figure 6), which may be one of the reasons for the higher level of *HOX* gene expression. Therefore, we can assume that the activation of the RA pathway, similarly to that during embryonic development, contributes to the abnormal activation of *HOX* genes at the initial stages of neuronal differentiation of iPSCs derived from PD patients [20].

It should be noted that the WNT/β-catenin pathway was considered one of the main pathways involved in PD, as was shown [51,52]. There are data on the direct regulation of the *HOXA* gene cluster expression by Wnt signaling [53]. In its turn, the RA via RARα can influence the Wnt/β-catenin pathway and additionally activate *HOX* gene transcription [39]. We suppose that in normal cells, this mechanism is not active, but in the cells of PD patients, it abnormally turns on. As was shown in [51], PARK2 protein is able to ubiquitinate β-catenin and limits its expression, while the *PARK2* gene mutations can lead to the accumulation and abnormal activity of β-catenin protein [51,52], which can lead to the *HOX* gene upregulation. In consistance with this hypothesis, we observed an elevated level of *WNT3* and *RARα* gene expression.

In its turn, the slightly activated expression level of 5′ *HOX* paralogs corresponds well with the downregulation of the *FGF8* gene (Figure 6), which is known as an activator of 5′ *HOX* paralogs.

Thus, we suppose that the observed dysregulated expression of 3′-HOX genes in the neuronal derivatives from PD patients appears to be caused by disturbance of RA-WNT/ β-catenin signaling; in particular, PARK2 inactivation can lead to an abnormal increase of the β-catenin quantity and subsequent enhanced HOX gene expression. It should be borne in mind that HOX proteins play the role of transcription factors that regulate the expression of many genes. HOX protein target genes are involved in the regulation of key processes in the cell (cell cycle, apoptosis, migration/invasion, inflammation, etc.) [54,55,56,57,58]. The abnormal activation of these processes can negatively influence the neuronal cells of PD patients. This indicates that in order to establish the cause of PD, one should most likely search not so much for individual genes associated with PD but pay attention to those metabolic processes that change in this disease.

When evaluating our data, we should keep in mind that modeling PD in iPSCs, as well as other existing models of this disease, has certain limitations despite many advantages [10,11]. PD is associated with aging, and it is a limitation of in vitro iPSC technology to generate an age-matched model to more fully recapitulate the PD phenotypes in vivo. Although we cannot consider the neuronal derivatives of iPSCs as a real model of adult human brain cells, our data on the *HOX* gene expression in HD cells are consistent with the current observation that *HOX* genes are transcribed at low levels in a limited subset of cells in healthy adult brains [20].

Summarizing, the data obtained showed that in the majority of PD patients in NPC and TDN, an increased expression of the *HOX* gene was observed. The contribution of *HOX* gene dysregulation to the development of PD remains enigmatic. *HOX*-related mechanisms associated with neurodegeneration require further study.

## Figures and Tables

**Table 1:** Description of cell lines used.

Designation	Description of PD Patients and HD	Genotype	NPC Cell Line Name	TDN Cell Line Name	Abbreviation Name of Cell Lines
IPSRG2L [12]	Healthy male, 60 years	normal	NP RG2L	TDN RG2L	HD1
IPSHD1.1S [12]	Healthy female, 18 years	normal	NP HD 1.1S	TDN HD 1.1S	HD2
IPSFD3.9L [12]	Healthy female, 26 years	normal	NP RFD 3.9 L	TDN RFD 3.9 L	HD3
PARK2-PDL1 [12]	Male with PD, the disease onset—38 years, biopsy—40 years	(del 202-203 AG: IVS1 + 1G/A) *PARK2*	NP PDL1.5L	TDN PLD1.5L	PD1
PSPDPS8 [12]	Female with PD, the disease onset—30 years, biopsy—41 years	EX8 del *PARK2*	NP PDS13	TDN PDS13	PD2
IPSPDPS2d [12]	Male with PD, the disease onset—38 years, biopsy—40 years	het EX2 del *PARK2*	NP PDS14	TDN PDS14	PD3
IPSPDL2.15L [32]	Male with PD the disease onset—50 years, biopsy—63 years	G2019S *PARK8*	NP PDL2.15L	TDN PDL2.15L	PD4
IPSPDG1.1S [32]	Male with PD the disease onset—44 years, biopsy—60 years	N370S *GBA*	NP PDG1.1S	TDN PDG1.1S	PD5
IPSPDL1.6L [32]	Male with PD, the disease onset—47 years, biopsy—58 years	G2019S *PARK8* N370S *GBA*	-	TDN PDL1.6L	PD6

## Data Availability

The data analyzed in this study are presented in GEO series GSE 181029.

## Supplementary Materials

The following supporting information can be downloaded at: https://www.mdpi.com/article/10.3390/jdb11020023/s1. Figure S1: Scheme of experiment; Figure S2: Immunocytochemical staining of NPCs; Figure S3: Immunocytochemical staining of TDNs; Figure S4: Transcription profiling (TPM) of *HOX* genes clusters in NPCs from PD2 and PD3 patients; Figure S5: Transcription profiling (TPM) of *HOX* genes clusters in TDNs from PD2 and PD3 patients; Figure S6: Heatmap of the HOX gene expression in fibroblasts (FB), iPSC, NPC, and TDN cell lines from PD and HD obtained using qPCR; Figure S7: NRC calculated in cells from discordant twins; Figure S8: Fragments of the HOXC-AS2 sequence corresponding to CTCF responsive elements; Table S1: The comparison of the expression of the marker genes in NPCs and TDNs from HD and PD patients; Table S2: GSEA results. HD TDNs vs. HD NP; Table S3: GSEA results. PD2 and PD3 TDNs vs. PD2 and PD3 NPs; Table S4: Gene name and ID (ENSEMBL); Table S5: Primers used in this study; Table S6: GSEA results of NPC and TDN.
