# Supplementary material for: Transcription of HOX Genes Is Significantly Increased during Neuronal Differentiation of iPSCs Derived from Patients with Parkinson’s Disease
Source: J Dev Biol. 2023 May 25;11(2):23. doi: 10.3390/jdb11020023 (PMC10299083; doi:10.3390/jdb11020023)
Supplement: Supplementary file 1 [file jdb-11-00023-s001.zip › jdb-2365607-supplementary.pdf]

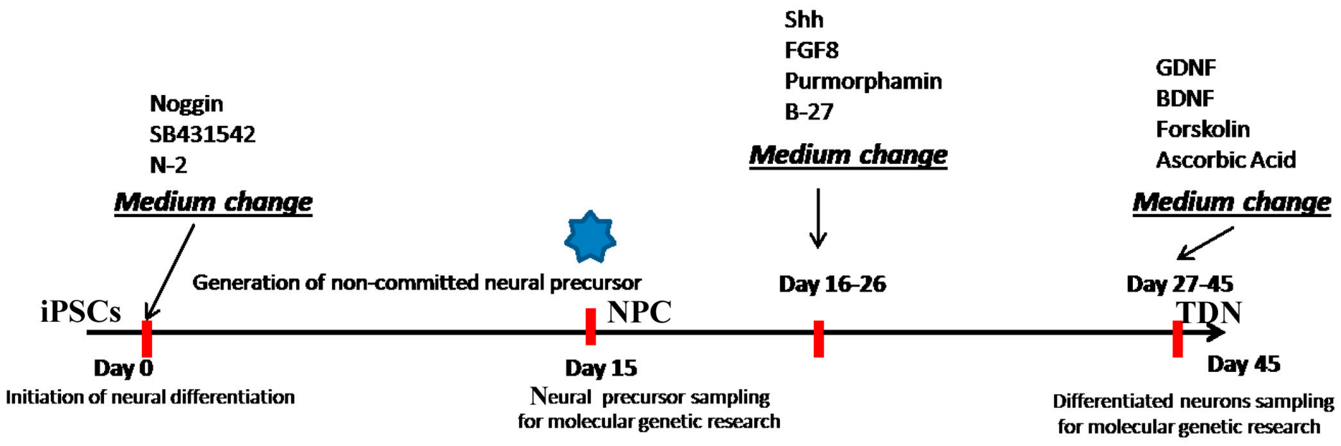

Figure S1. Scheme of experiment.

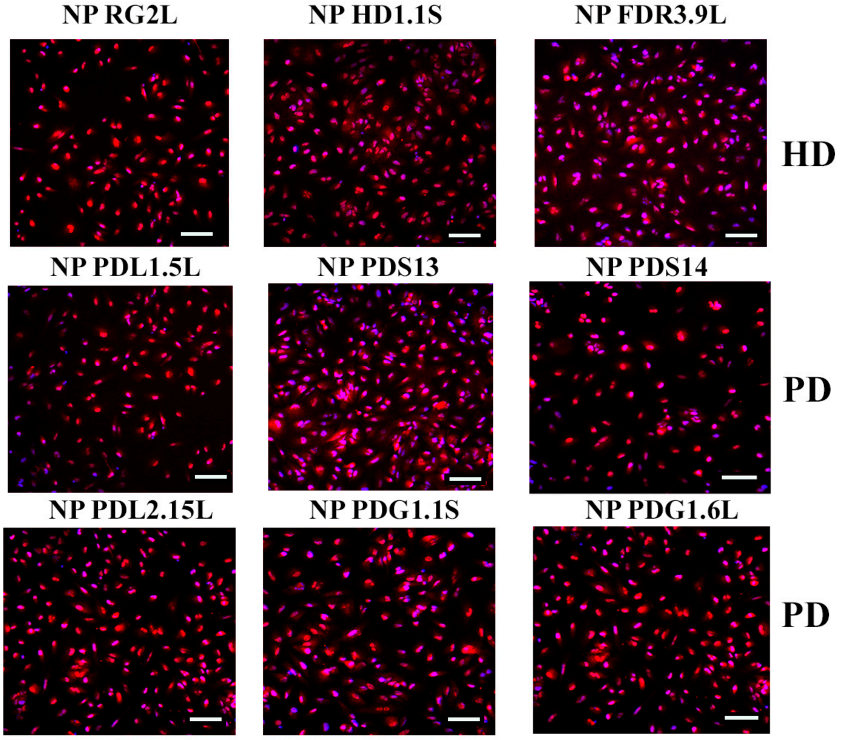

Figure S2. Immunocytochemical staining of NPCs with antibodies against Sox1 (red) and nuclear dye DAPI (blue), magnification X 100. NP RG2L (HD1), NP HD 1.1S(HD2), NP RFD 3.9 L(HD3), NP PDL1.5L(PD1), NP PDS13(PD2), NP PDS14(PD3), NP PDL2.15L (PD4), NP PDG1.1S (PD5), NP-PDG 1.6S (PD6).

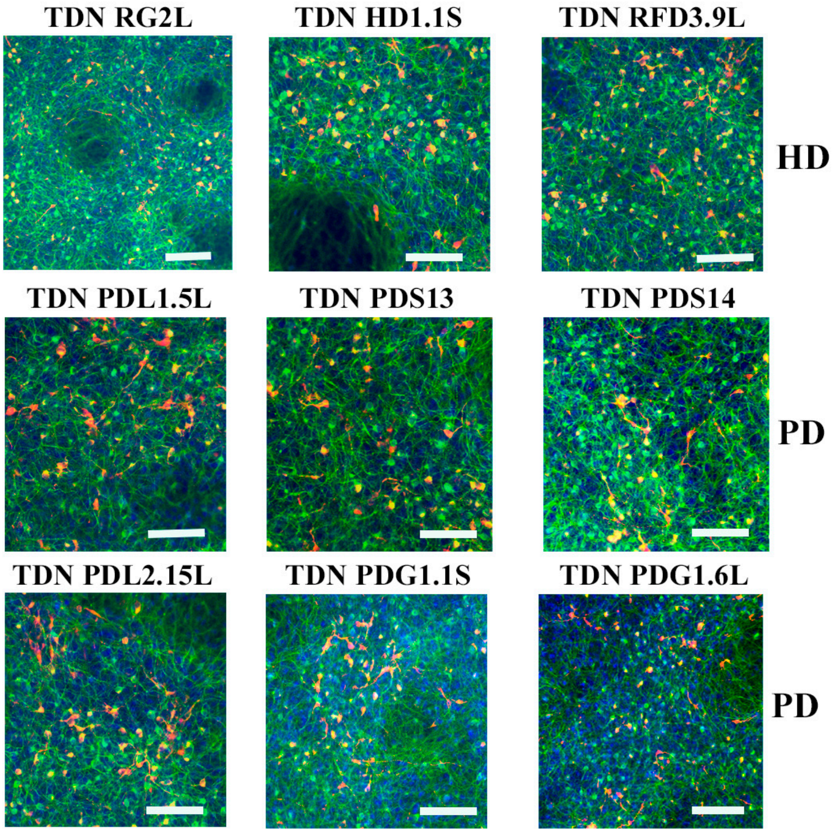

Figure S3. Immunocytochemical staining of TDNs with antibodies against β III tub (green), tyrosine hydroxylase (red) and nuclear dye DAPI (blue), magnification X 100. TDN RG2L(HD1), TDN HD 1.1S(HD2), TDN RFD 3.9 L(HD3), TDN PDl1.5L(PD1), TDN PDS13(PD2), TD NPDS14(PD3), TDN PDL2.15L (PD4), TDN PDG1.1S (PD5), TDN-PDG 1.6S (PD6).

Table S1. The comparison of the expression of the marker genes in NPCs and TDNs from HD and PD patients.

	TDN/NPC	
Genes	HD	PD
NESTIN	0.88	0.82
PAX6	1.05	0.23
TUBB3	4.47**	2.49**
NEUN	8.6**	6.47**
MAP2	1.56**	8.95**
MSI1	1.92**	1.94**
SNAP25	1.56**	3.77**
SYT1	8.39**	4.24**
PSD95	5.12**	3.03**
RAB5A	2.7**	1.75**
NURR1	7.41**	7.11**
VMAT2	1.76**	1.43

* $p < 0.05$ and ** $p < 0.01$ TDN vs. NPC according to two factor ANOVA test,

Table S2. GSEA results. HD TDNs vs. HD NP. Thresfold |Log_2(FC)| >2 and Pval<0.05.

**GO category**	**NES**	**FDR**	**Pval**
GO Molecular Function category			
GO:0030594 Neurotransmitter receptor activity	2.55	2.2E-16	2.2E-16
GO:0046783 Metal ion transmembrane transporter activity	2.42	2.2E-16	2.2E-16
GO:0022803 Passive transmembrane transporter activity	2.32	2.2E-16	2.2E-16
GO:0015077 Monovalent inorganic cation transmembrane transporter activity	2.30	0.00006	2.2E-16
GO:0000149 SNARE binding	2.09	0.0007	2.2E-16
GO:0030276 Clathrin binding	1.98	0.0042	2.2E-16
GO:0005326 Neurotransmitter transporter activity	1.91	0.009	0.0012
GO:0005112 Notch binding	1.84	0.02	0.003

NES, normalized enrichment score, FDR, false discover rate

Table S3. GSEA results. PD2&PD3 TDNs vs. PD2&PD3 NPs. Thresfold $|Log_2(FC)| > 2$ and Pval<0.05.

GO Category	NES	FDR	Pval
GO Molecular Function category			
GO:0030594 Neurotransmitter receptor activity	2.86	2.2E-16	2.2E-16
GO:0008066 Glutamate receptor activity	2.69	2.2E-16	2.2E-16
GO:0042165 Neurotransmitter binding	2.54	2.2E-16	2.2E-16
GO:0046873 Metal ion transmembrane transporter activity	2.37	0.00004	2.2E-16
GO:0022803 Passive transmembrane transporter activity	2.32	0.00006	2.2E-16
GO:0030276 Clathrin binding	2.29	0.00008	2.2E-16
GO:0005326 Neurotransmitter transporter activity	2.1	0.0016	2.2E-16
GO:0030551 Cyclic nucleotide binding	2.09	0.0016	0.00044

NES, normalized enrichment score, FDR, false discovery rate

Table S4. Gene name and ID (ENSEMBL)

Cluster	Gene Name (coding)	ID (ENSEMBL)	Gene Name (lncRNA)	ID (ENSEMBL)
HOXA	*HOXA1*	ENSG00000105991	*HOTAIRM1*	ENSG00000233429
	HOXA2	ENSG00000105996	*HOXA-AS2*	ENSG00000253552
	HOXA3	ENSG00000105997	*HOXA-AS3*	ENSG00000254369
	HOXA4	ENSG00000197576	*AC004080.6*	ENSG00000273433
	HOXA5	ENSG00000106004	*AC004080.5*	ENSG00000272801
	HOXA6	ENSG00000106006	*AC004080.4*	ENSG00000270182
	HOXA7	ENSG00000122592	*HOXA10-AS*	ENSG00000253187
	HOXA9	ENSG00000078399	*HOXA11-AS*	ENSG00000240990
	HOXA10	ENSG00000253293	*HOTTIP*	ENSG00000243766
	HOXA11	ENSG00000005073		
HOXB	*HOXB1*	ENSG00000120094	*HOXB-AS1*	ENSG00000230148
	HOXB2	ENSG00000173917	*HOXB-AS2*	ENSG00000239552
	HOXB3	ENSG00000120093	*HOXB-AS3*	ENSG00000233101
	HOXB4	ENSG00000182742	*HOXB-AS4*	ENSG00000242207
	HOXB5	ENSG00000120075	*AC103702.1*	ENSG00000257178
	HOXB6	ENSG00000108511	*AC103702.2*	ENSG00000272763
	HOXB7	ENSG00000260027	*LINC02086*	ENSG00000244649
	HOXB8	ENSG00000120068		
	HOXB9	ENSG00000170689		
	HOXB13	ENSG00000159184		
HOXC	*HOXC4*	ENSG00000198353	*HOXC-AS1*	ENSG00000250451
	HOXC5	ENSG00000172789	*HOXC-AS2*	ENSG00000250133
	HOXC6	ENSG00000197757	*HOXC-AS3*	ENSG00000251151
	HOXC8	ENSG00000037965	*LINC02381*	ENSG00000250742
	HOXC9	ENSG00000180806	*FLJ12825*	ENSG00000248265
	HOXC10	ENSG00000180818	*AC012531.1*	ENSG00000260597
	HOXC11	ENSG00000123388	*RP11-834c11.14*	ENSG00000273046
	HOXC12	ENSG00000123407	*AC023794.2*	ENSG00000249388
	HOXC13	ENSG00000123364		
HOXD	*HOXD1*	ENSG00000128645	*HOXD-AS1(HAGRL)*	ENSG00000224189
	HOXD3	ENSG00000128652	*HOXD-AS1(HAGRLOS)*	ENSG00000226363
	HOXD4	ENSG00000170166	*HOXD-AS2*	ENSG00000237380
	HOXD8	ENSG00000175879	*AC009336.1*	ENSG00000272729
	HOXD9	ENSG00000249499		
	HOXD10	ENSG00000128710		
	HOXD11	ENSG00000128713		
	HOXD12	ENSG00000170178		
	HOXD13	ENSG00000128714		

Table S5. Primers used in this study

Gene	Primer sequences (forward/reverse)
18s rRNA	F: CGGCTACCACATCCAAGGAA R: GCTGGAATTACCGCGGCT
HOXA3	F: AGCTCCAGCTCAGGCGAAAG R: TAGCGGTTGAAGTGGAACTCTT
HOXA5	F: GCTGCACATAAGTCATGACAAC R: AGGTAACGGTTGAAGTGGAACT
HOXA7	F: GGAGTTCCACTTCAACCGCTA R: CAGTCGGACCTTCGTCCTTATG
HOXA9	F: GGCGCCTTCTCTGAAAACAATG R: GTTGGCTGCTGGGTTTTGGGA
HOXB3	F: ATGCAGAAAGCCACCTACTACG R: CTGCCAGGGTACGAGGAATAG
HOXB8	F: TCGCAAATCCAGGAGTTCTACC R: CGGGTCGTAGCCGTAGAAATTG
HOXB9	F: GAGAGGCCGGATCAAACCAAC R: CTAGCTCCAGCGTCTGGTATTT
HOXC4	F: TCACGTTAGCACGGTGAACCCC R: CTTCTCCTTCGGGTCAGGTAGC
HOXC5	F: GCACATGAGCCACGAGACGG R: GCGAGTGAGGTAGCGGTTAAAG
HOXC6	F: GAATTCGCACAGTGGGGTCGG R: CTGGTACCGCGAGTAGATCTGG
HOXD3	F: ATGCTTCTAGCTCCTCAGCCAC R: GTCCTCGCAGCTCTCTCCTGC
HOXD8	F: TGGATGAGACCACAAGCAGCTC R: CTCTAGAGTTTGGAAGCGACTGT

Table S6. GSEA results (threshold |Log_2(FC)|>2 and Pval<0.05).

NPC (PD2&PD3 vs HD)	Category	NES	FDR	Pval
**GO: Molecular Function**				
GO:0035326	Enhancer binding	2.30	2.2E-16	2.2E-16
GO:0001228	DNA-binding transcription activator activity, RNA polymerase II-specific	2.09	0.00013	2.2E-16
GO:0033613	Activating transcription factor binding	1.99	0.001	2.2E-16
**GO: Biological Process**				
GO:0048706	Embryonic skeletal system development	2.66	2.2E-16	2.2E-16
GO:0021510	Spinal cord development	2.41	2.2E-16	2.2E-16
GO:0048705	Skeletal system morphogenesis	2.37	2.2E-16	2.2E-16
GO:0007389	Pattern specification process	2.30	2.2E-16	2.2E-16
GO:0048568	Embryonic organ development	2.22	2.2E-16	2.2E-16
GO:0048736	Appendage development	2.12	0.00005	2.2E-16
GO:0021675	Nerve development	2.09	0.00009	2.2E-16
GO:0035270	Endocrine system development	2.09	0.00008	2.2E-16
GO:0045165	Cell fate commitment	2.01	0.0006	2.2E-16
GO:0031016	Pancreas development	1.98	0.0013	2.2E-16
**Reactome**				
R-HSA-5617472	Activation of anterior HOX genes in hindbrain development during early embryogenesis	2.32	2.2E-16	2.2E-16
R-HAS-5619507	Activation of HOX genes during differentiation	2.32	2.2E-16	2.2E-16
**TDN (PD2&PD3 vs HD)**				
**GO: Molecular function**				
GO:0035326	Enhancer binding	2.61	2.2E-16	2.2E-16
GO:0001228	DNA-binding transcription activator activity, RNA polymerase II-specific	2.53	2.2E-16	2.2E-16
**GO: Biological Process**				
GO:0048706	Embryonic skeletal system development	2.86	2.2E-16	2.2E-16
GO:0007389	Pattern specification process	2.68	2.2E-16	2.2E-16
GO:0048705	Skeletal system morphogenesis	2.59	2.2E-16	2.2E-16
GO:0021675	Nerve development	2.32	0.0005	2.2E-16
GO:0048568	Embryonic organ development	2.12	0.006	2.2E-16
**Wikipathway**				
WP2855	Dopaminergic Neurogenesis	1.98	0.03	0.0007

NES, Normalized enrichment score, FDR, False discovery rate

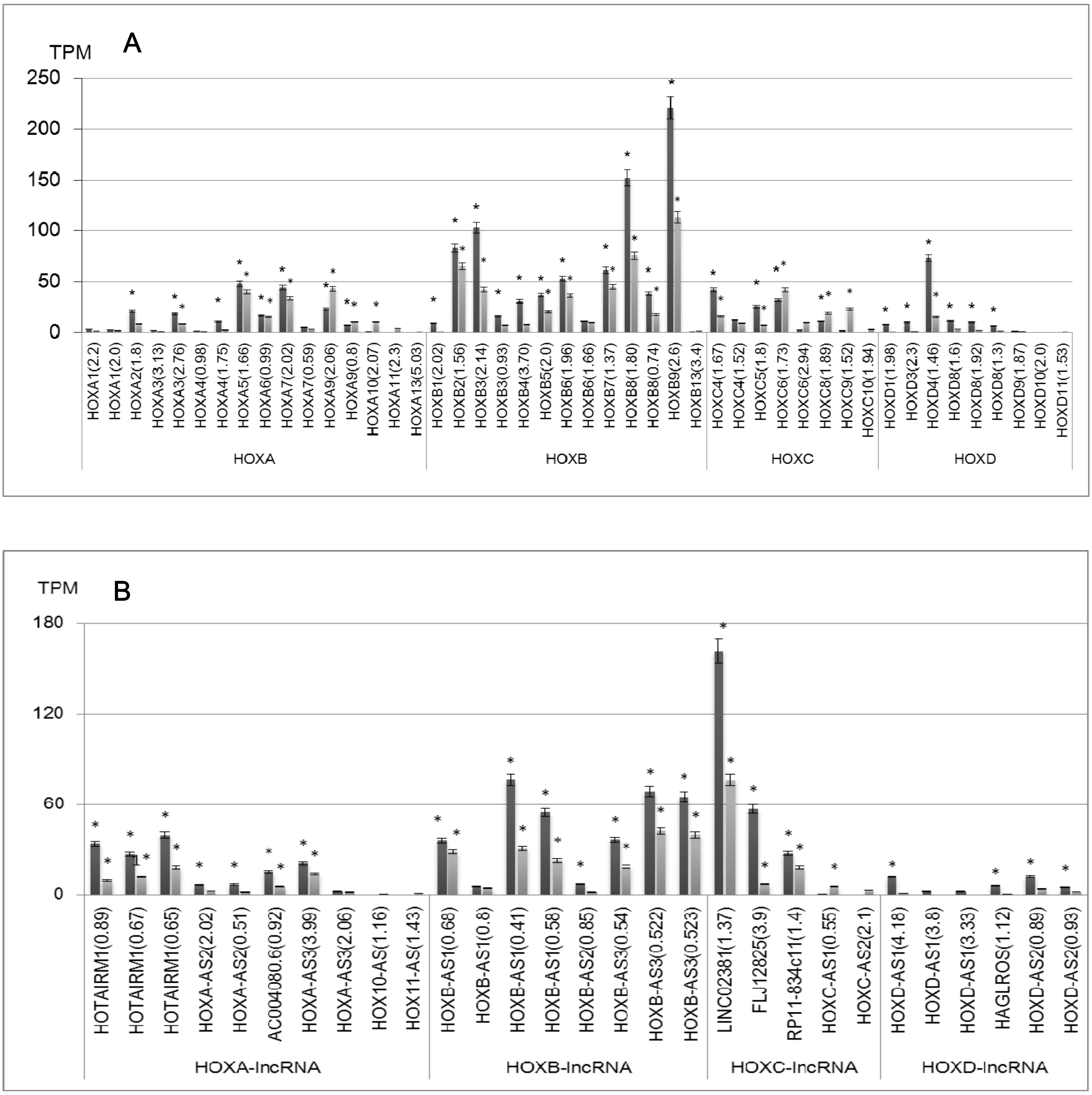

Figure S4. Transcription profiling of protein-coding (**A**) and lncRNA (**B**) genes of *HOX* clusters in NPCs from PD2 patient (black) and PD3 patient (grey) expressed as the number of TPM for transcript variants. The order of the genes corresponds to their location in the cluster in the 3' to 5' direction. The length of mRNA variants in kilobases is indicated in parentheses. Statistical significance is estimated by Manna-Whitney U-test (* $P<0.05$ for PD cells vs. HD cells).

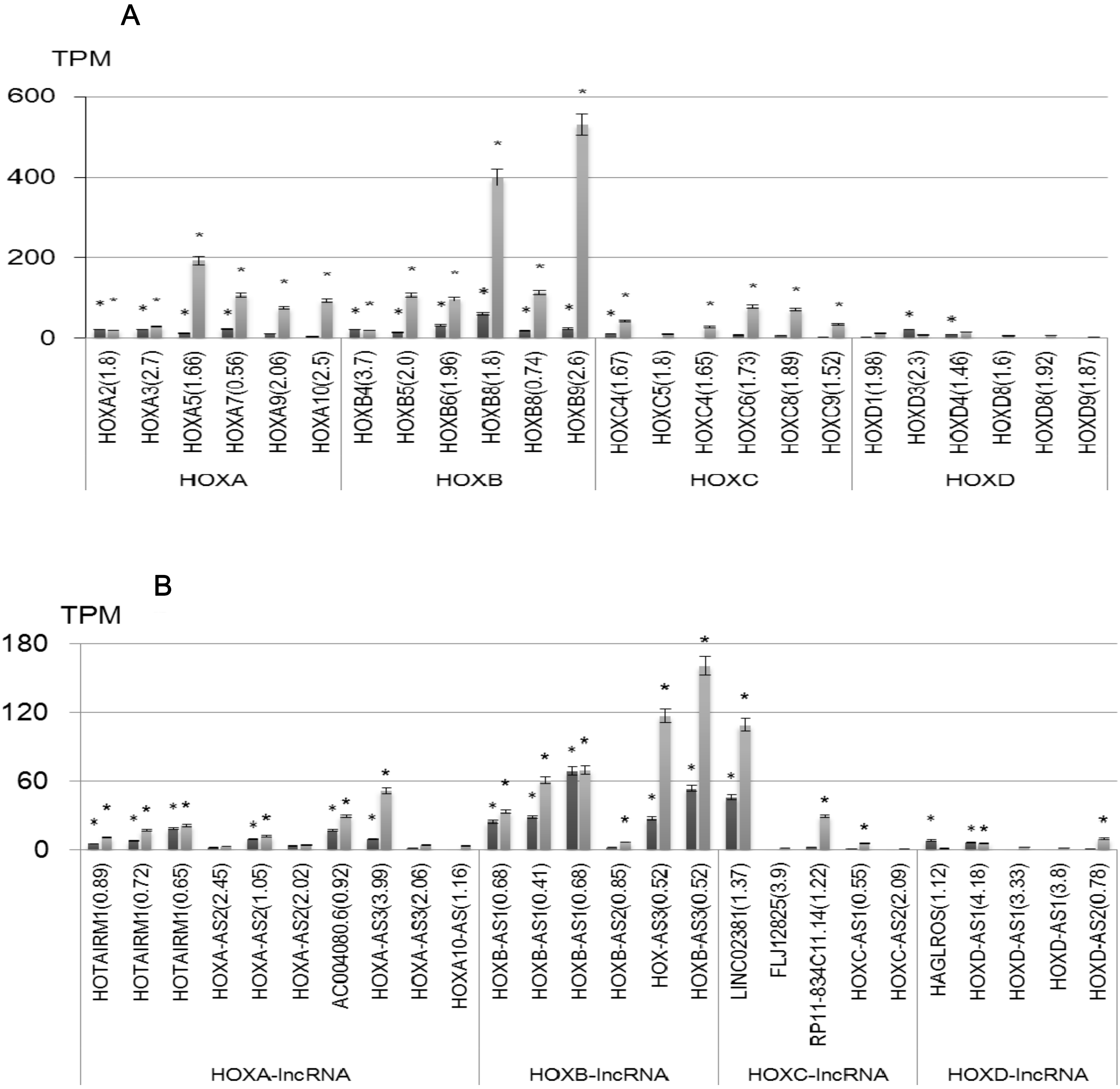

Figure S5. Transcription profiling of protein-coding (**A**) and lncRNA (**B)** genes of *HOX* clusters in iPSC-derived TDN from PD2 (black) and PD3 (grey) patient, which expressed as the number of TPM for transcript variants. The order of the genes corresponds to their location in the 3' to 5' direction in the cluster. The length of mRNA in kilobases is indicated in parentheses. Statistical significance is estimated by Manna-Whitney U-test (* P<0.05 for PD cells vs. HD cells).

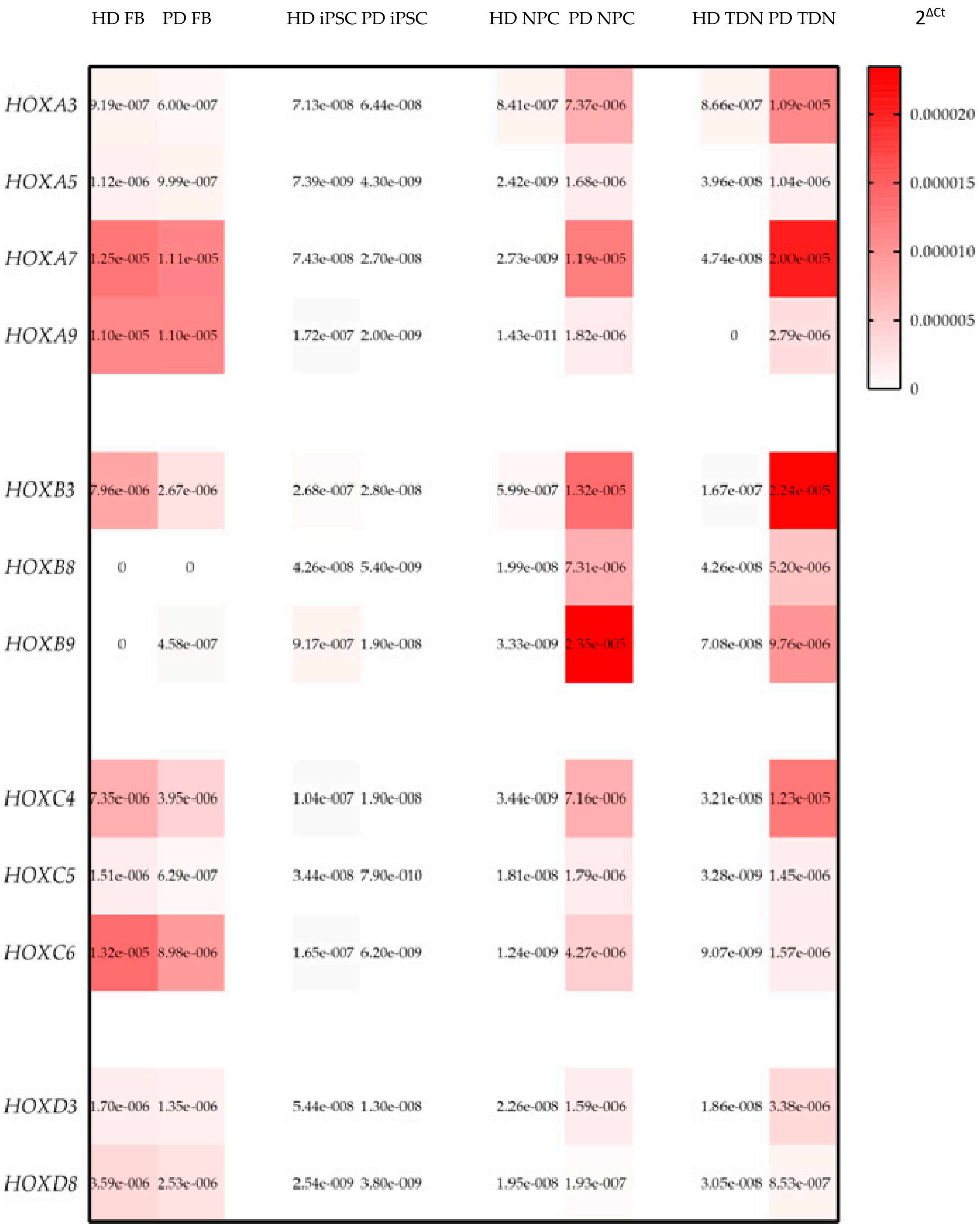

Figure S6. Heatmap of the *HOX* gene expression in fibroblasts (FB), iPSC, NPC, and TDN cell lines from PD (mean of PD2, PD3, PD4, PD5, and PD6) and HD (mean of HD1, HD2, HD3) obtained using qPCR. The data were normalized

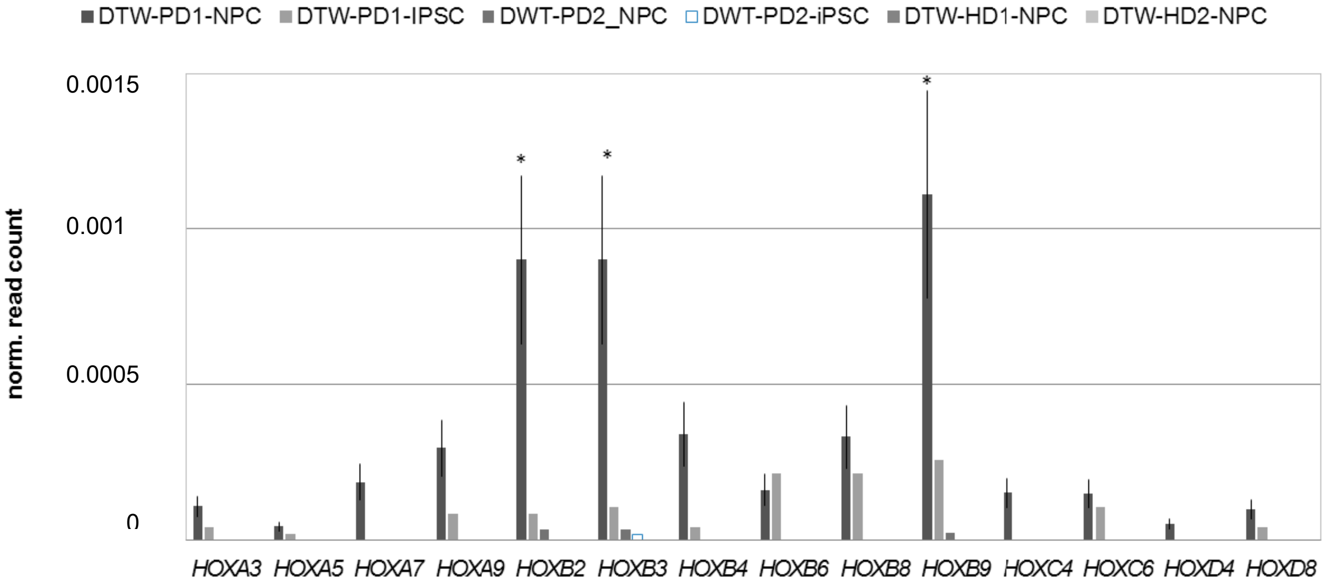

Figure S7. NRC calculated in cells from discordant twins (DTW) (GSE185009). The transcription of HOX genes is up-regulated in NPC from patient with PD (DTW-PD1-NPC) compairing with that in DTW-PD1-iPSC, DTW-PD2-NPC, DTW-PD2-iPSC, DTW-HD1-NPC, DTW-HD2-NPC. Manna-Whitney U-test was used. *$P<0.05$.

361 tcttagagtt **<u>ccctc</u>**ccacc cccaaaagac ttcactgggc acaggagaga cccctgaggg

481 caccgtggga aggacagaca gaaagataga caggttagag tcgtc**<u>ccctc</u>** cccctgcccc

781 tccccgcc**<u>cc</u>** **<u>ctc</u>**cctggtt c**<u>ccctc</u>**tact ttcccccctt ctctctcctg at**<u>ccctc</u>**ttt

1921 agccccggcc accacggccg ccgccgcccg **<u>ccctc</u>**ctt **<u>ccctccctccct c</u>**cttctcg **<u>cc</u>**

1981 **<u>ctccctc</u>**tct ct**<u>ccctc</u>**cct cgcgcgctgc cgctcgcggg g**<u>ccctc</u>**ccgc gggcaacctg

Figure S8. Fragments of the HOXC-AS2 sequence with underlined bold letters corresponding to CTCF sensitive elements. Start point location is chromosome 12: 53993810 - 53996785 in reverse orientation GRCh38.
